# Scrutinizing MHC-I Binding Peptides and Their Limits of Variation

**DOI:** 10.1371/journal.pcbi.1003088

**Published:** 2013-06-06

**Authors:** Christian P. Koch, Anna M. Perna, Max Pillong, Nickolay K. Todoroff, Paul Wrede, Gerd Folkers, Jan A. Hiss, Gisbert Schneider

**Affiliations:** 1 ETH Zürich, Department of Chemistry and Applied Biosciences, Institute of Pharmaceutical Sciences, Zürich, Switzerland; 2 Charite-Universitätsmedizin Berlin, Molekularbiologie und Bioinformatik, Berlin, Germany; University of Houston, United States of America

## Abstract

Designed peptides that bind to major histocompatibility protein I (MHC-I) allomorphs bear the promise of representing epitopes that stimulate a desired immune response. A rigorous bioinformatical exploration of sequence patterns hidden in peptides that bind to the mouse MHC-I allomorph H-2K^b^ is presented. We exemplify and validate these motif findings by systematically dissecting the epitope SIINFEKL and analyzing the resulting fragments for their binding potential to H-2K^b^ in a thermal denaturation assay. The results demonstrate that only fragments exclusively retaining the carboxy- or amino-terminus of the reference peptide exhibit significant binding potential, with the N-terminal pentapeptide SIINF as shortest ligand. This study demonstrates that sophisticated machine-learning algorithms excel at extracting fine-grained patterns from peptide sequence data and predicting MHC-I binding peptides, thereby considerably extending existing linear prediction models and providing a fresh view on the computer-based molecular design of future synthetic vaccines. The server for prediction is available at http://modlab-cadd.ethz.ch (SLiDER tool, MHC-I version 2012).

## Author Summary

Future success in vaccine development will critically depend on identifying potent epitopes with reduced side effects. Among such candidate molecules, immunogenic peptides binding to major histocompatibility protein I (MHC-I) represent a preferred class of biomolecules for vaccine design. Computational models assist in the selection of the best candidate peptides by providing a mathematical rationale for antigen recognition by MHC-I. Here we present a machine-learning model that was trained on recognizing features of known MHC-I binding and non-binding peptide sequences with sustained accuracy. We were able to biochemically validate the computational predictions in a direct binding assay measuring complex formation between synthesized candidate peptides and MHC-I. Strong correspondence between the predictions and the experimentally determined binding potential corroborate the machine-learning model as viable for future antigen design. Thus, our study provides a concept for rapidly finding innovative MHC-I binding peptides with limited experimental effort.

## Introduction

**Figure 1:**
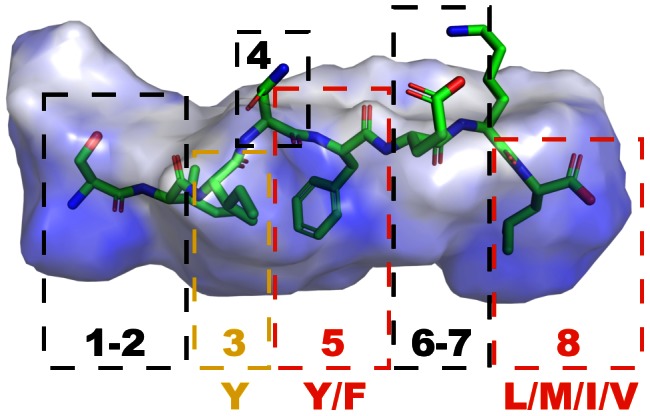
Volume representation of the MHC H-2K^b^ peptide binding cleft derived from a crystal structure model (PDB-ID [**63**]: 1vac [**64**]) with the protein-bound epitope SIINFEKL. Numbers within dashed boxes correspond to sequence positions in the respective epitope. Red boxes and associated amino acid codes indicate anchor positions and preferred amino acid composition respectively. The yellow box indicates the secondary anchor at position 3, according to the H-2K^b^ canonical sequence motif.

Artificial induction of immunity (immunization) is achieved by priming the immune system with a specific antigen (epitope) bearing the potential of activating the adaptive immune response [1]. Vaccines are often synchronously administered with adjuvants aiming at boosting the adaptive immune system by activation of the innate immune system through pattern recognition receptors [2], [3]. Aside from introducing excessive non immune-stimulating pathogenic material into the patient, drawbacks of all clinically applied vaccines include poor or incomplete inactivation, reversion of virulence and limitation to viral pathogens [4]. Utilizing synthetic peptides as antigen mimetics bear the promise to avert some of these effects [5]–[7]. The importance of prediction and design of major histocompatibility protein I (MHC-I) bound peptides that are recognized as a complex by receptors on cytotoxic T cells (cell-mediated immunity) was outlined by Rammensee and co-workers in 1993, who defined canonical sequence motifs for a set of MHC-I alleles (Fig. 1) [8]–[10]. Residue patterns emerged from a statistical assessment of the frequency and effects of certain amino acids at specified epitope positions. In the subsequent two decades, several prediction models were established for various MHC-I alleles from different organisms, taking into account not only isolated residue positions and their amino acid occupancy but also their positional correlation [11]. Maximizing the predictive accuracy of these models by using modern machine-learning approaches such as cascaded [12] or deep-learning [13] architectures bears the chance of increasing the probability of successfully designing synthetic epitopes, acknowledging that MHC-I binding potential poses a necessary prerequisite yet not a guarantee for inducing an immune response.

In this study we developed a cascaded machine learning approach to learn patterns from the available MHC-I binding information in the Immune Epitope Database (IEDB) [14] regarding the murine H-2K^b^ allele. We selected this allele and binding octapeptides due to the allele's character as reference model and data availability. Using our new predictive model, we investigated the sequence variability of the well-studied ovalbumin-derived epitope SIINFEKL [15]. We scrutinized octapeptides containing SIINFEKL fragments, while actual fragments were, in contrast to earlier surface plasmon resonance studies [16] or fluorescence labeling [15], [17], tested in a thermal denaturation assay [18] for their direct binding potential to an H-2K^b^/IgG fusion protein. Formerly understood patterns of epitope binding to the H-2K^b^ allele were described in form of Rammensee's statistically derived canonical sequence motif (Fig. 1) [8], [9], which consists of a tyrosine or phenylalanine residue at position 5 and an aliphatic amino acid at position 8 as so-called ‘main anchors’ as well as a tyrosine at position 3 as ‘secondary anchor’: (3[Y]-5[Y,F]-8[L,M,I,V]). As a result of our study this generic concept was substantially extended with our machine-learning model and validated with rapid direct-binding experiments, which ideally complement strenuous and resource-intensive cell-based assays [19], [20]. In contrast to the utilization of peptide libraries, the computational approach offers a comprehensive alternative for the analysis of epitope binding motifs with minimal experimental effort involved, thereby additionally focusing on varying fragment lengths and related requirements for binding.

## Results

### Cascaded machine-learning model

**Figure 2:**
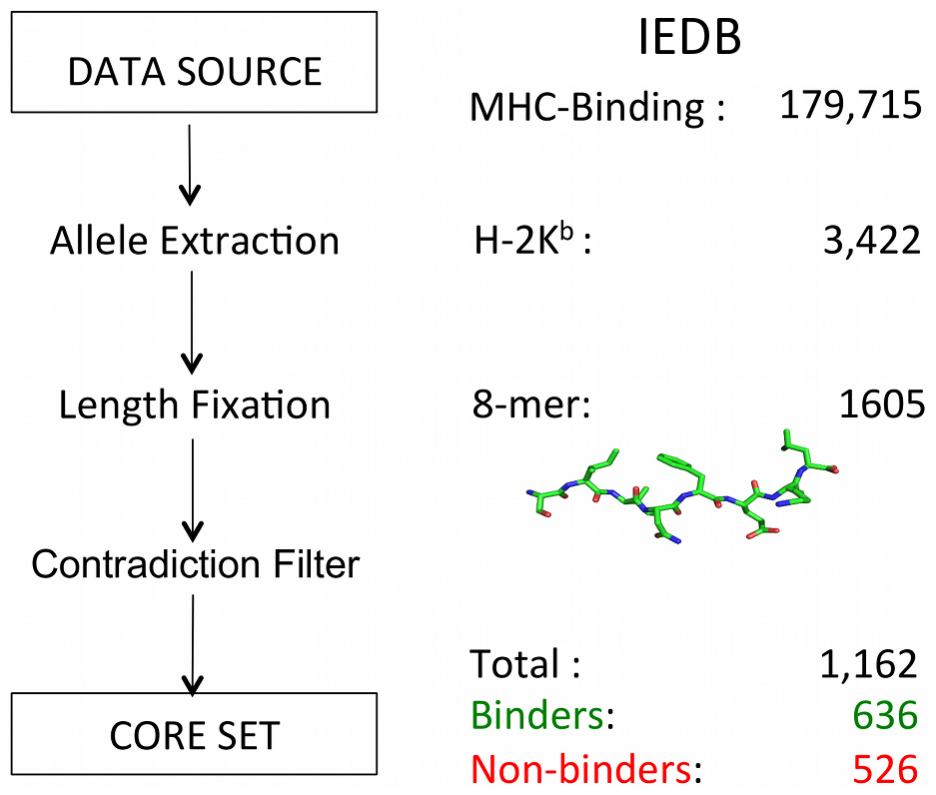
Training data derivation. The Immune Epitope Database (IEDB) served as data source [14]. The murine H-2K^b^ allele and a length of eight were selected due to good data availability and reference model character. The core set exhibits similar contributions of positive (636) and negative (526) examples, while redundant ambiguous entries were removed. The core set contained binders and non-binders partially, fully and not agreeing with the canonical sequence motif.

We extracted peptide data from version 7/2012 of the IEDB [14] to train an ensemble machine-learning model utilizing a cascaded architecture. After selecting octapeptides with binding information on the mouse H-2K^b^ allele (Fig. 2), a filter was applied draining ambiguous entries resulting in a core set of 1,162 peptides. Binders and non-binders were approximately balanced in this core data set (636 binders, 526 non-binders). We considered these sequence data as diverse due to the abundance of canonical motif complying and non-complying samples. Thus, the observation of often misleading performance estimates due to training data redundancy perceived in a study for MHC-II binding site prediction should not apply to our model [21].

**Figure 3:**
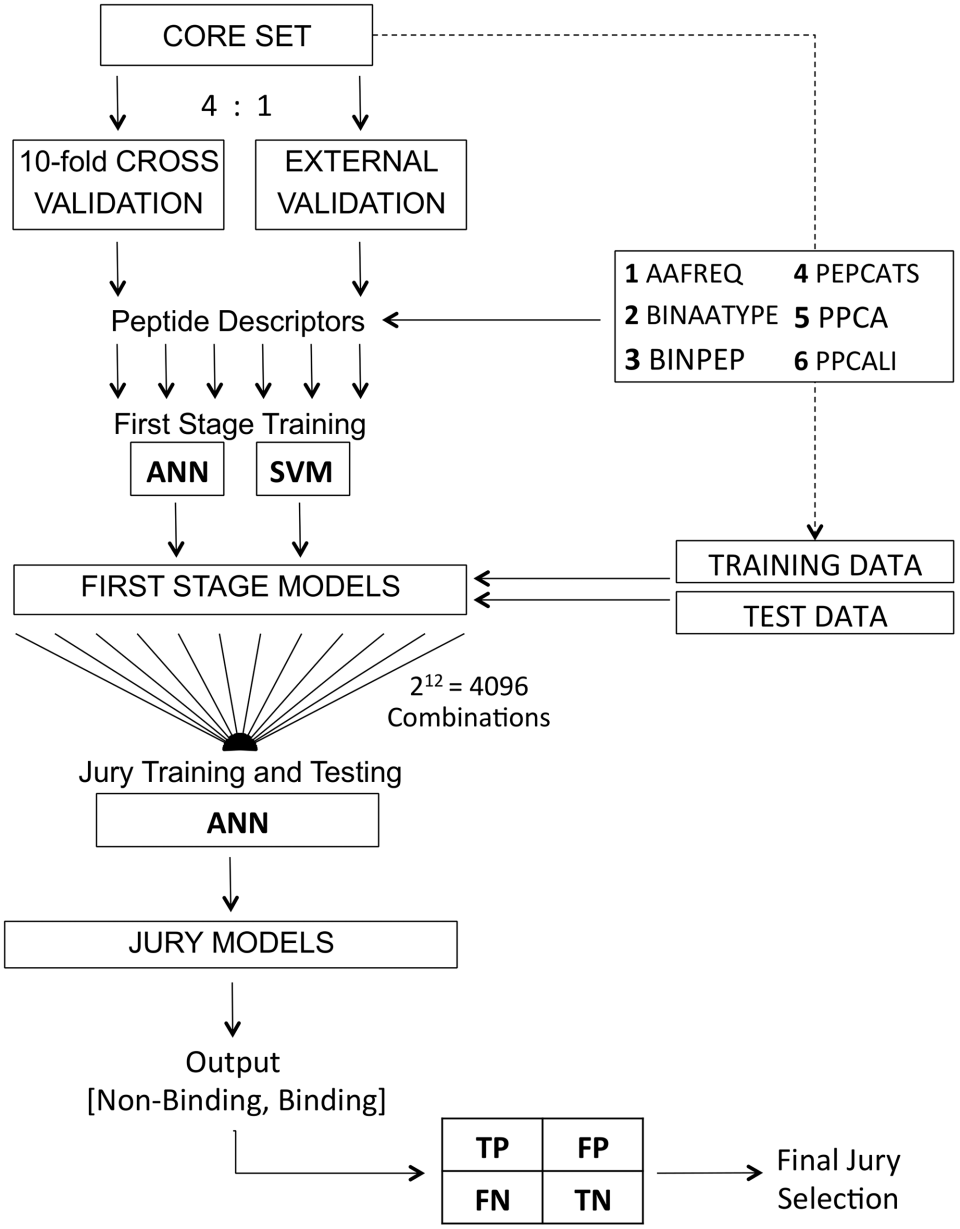
Workflow for the development of the cascaded machine-learning model. ANN: feed-forward artificial neural network, SVM: support vector machine. AAFREQ, BINAATYPE, BINPEP, PEPCATS, PPCA and PPCALI correspond to the utilized peptide descriptors (*cf.* Methods). TP, FP, FN and TN correspond to entities of a confusion table with true-positives, false-positives, false-negatives and true-negatives.

The core training set was split into a 10-fold cross-validation set and an external validation set by a ratio of 4∶1 in order to retain two evaluation scenarios (Fig. 3) [22]. Six amino acid descriptors, namely AAFREQ, BINAATYPE, BINPEP, PEPCATS, PPCA and PPCALI were calculated for every respective training fold. Two classifier models, multilayer Perceptron artificial neural networks [23] (ANN) and support vector machines [24] (SVM), were utilized for auto-parameterized first stage training resulting in a total of 6×2 = 12 first stage models (base classifiers) for every respective evaluation fold. All first stage models were subjected to implicit parameterization employing a grid-based five-fold cross-validation approach during training, utilizing online backpropagation of errors for ANNs [25] and a SMO-type [26] decomposition method for SVMs as training algorithms. ANNs were parameterized with respect to the number of neurons *h* ∈ [2,10] in the hidden layer, the learning rate η ∈ [0.1,1.0] and the momentum α ∈ [0.1,1.0], whereas SVMs were parameterized with respect to the cost parameter *C* ∈ [0.5,8.0] and kernel specific parameters degree *d* ∈ [1,3], γ ∈ [0.01,0.10] and *coef0* ∈ [0,6].

The 12 trained base classifiers were subsequently fed with the same data they were originally trained on to compute the input (*i.e.*, output neuron values from ANN or probability estimates from SVM; from the interval [0,1[) for the second-stage or ‘jury’ ANN classifier. We tested all possible input combinations, resulting in 2^12^ = 4096 jury neural networks, where each training instance for a jury ANN had as many dimensions as first stage classification models included. Test data was then presented to the trained first stage models for prediction and the fired output values were propagated to the according trained jury networks, which finally delivered a predicted output class (binder/non-binder; *score threshold* = 0.5). The output class prediction for every test data instance was compared to actual class labels in the test data and summarized in confusion tables (true-positives, false-positives, true-negatives, false-negatives). Confusion tables were then mapped into a single classification performance index (Matthews' correlation coefficient, *MCC*) [27] and the best performing final jury as a compromise of cross-validated and external set performance was selected and retrained on the entire core data.

**Figure 4:**
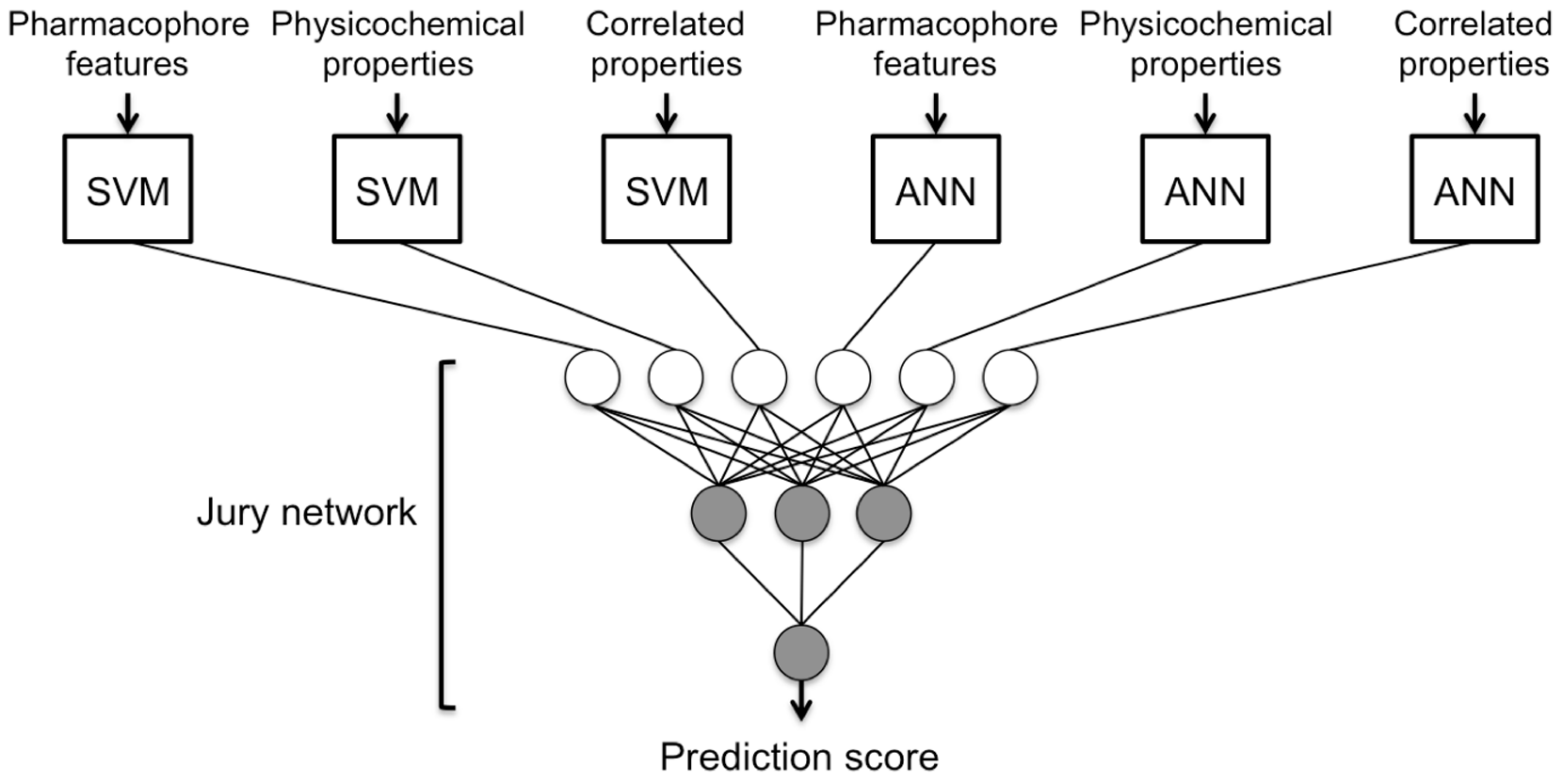
Architecture of the best-performing cascaded machine-learning model based on six first stage classifiers originating from three differing descriptor sets and two learning schemes (ANNs, SVMs) and a jury neural network containing three hidden neurons. The model delivers a prediction score from the interval [0,1[, with high values indicating MHC-I H-2K^b^ binding.

The final jury (Fig. 4) model (*MCC*_cross-validated_ = 0.64, *MCC*_external-set_ = 0.65) was selected and re-trained on the entire training data set. The obtained *MCC* of 0.64 is well within the quality of current state-of-the-art prediction tools for MHC-I prediction, and with regard to the cross-validated *MCC* suggests that jury over-training/over-fitting was avoided. The prediction method is publicly available at http://modlab-cadd.ethz.ch (SLiDER tool, MHC-I version 2012).

### *In silico* MHC-I binding prediction

**Figure 5:**
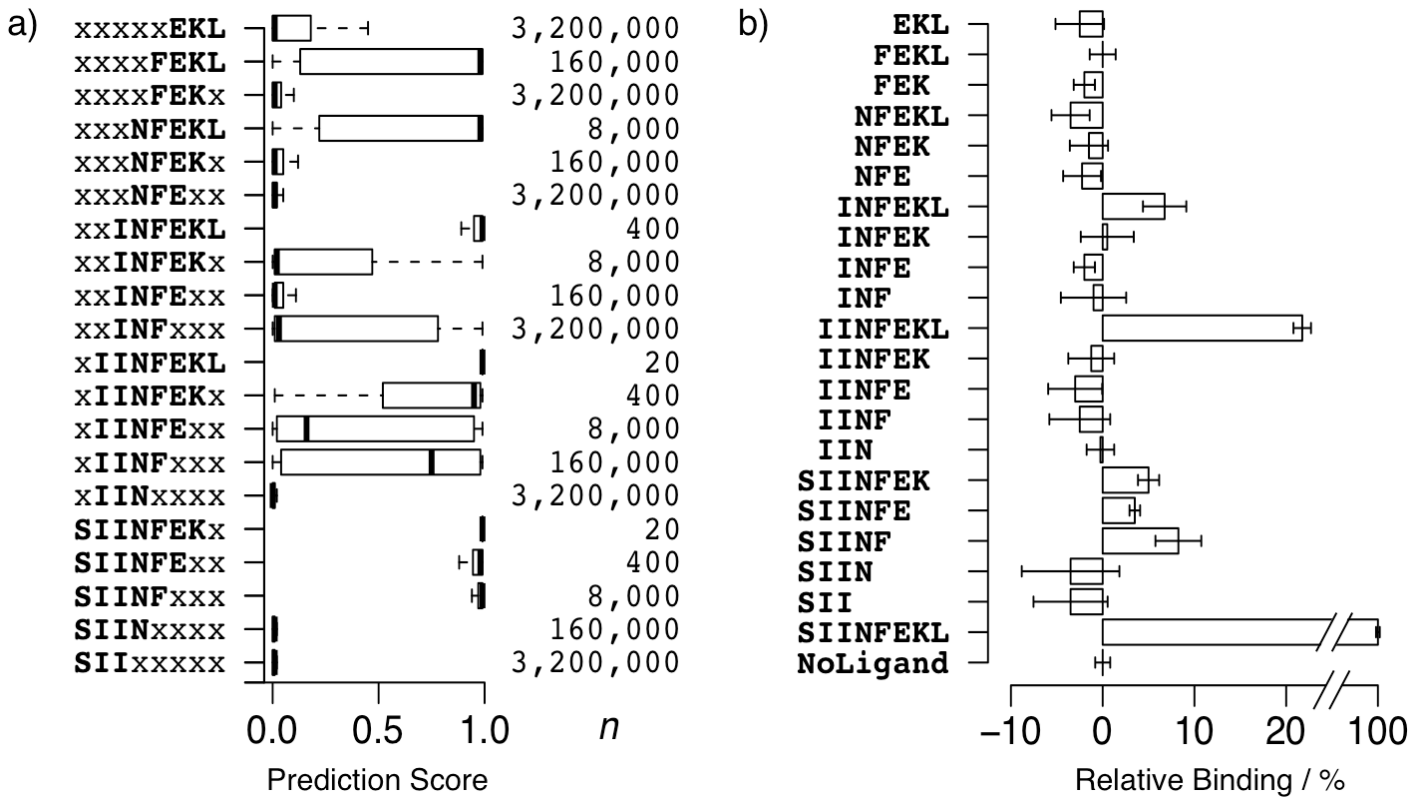
(**a**) Distribution of prediction scores for sets of *n* SIINFEKL-fragment containing octapeptides. High scores indicate MHC-I H-2K^b^ binding predictions, while lower scores indicate non-binding predictions. Scores were computed with the final jury prediction model. (**b**) Binding of synthesized SIINFEKL-fragments to a MHC-I H-2K^b^:IgG1 fusion protein relative to the average binding of the positive reference (SIINFEKL, 100%) and the unloaded fusion protein (*NoLigand*, 0%). Bars correspond to the arithmetic mean of quadruplicate measurements, with error whiskers depicting the volatility in terms of standard deviation.

All octapeptides containing at minimum tripeptide fragments of the positive reference binder SIINFEKL were categorically grouped according to the respective fragment (Fig. 5a). Resulting groups were classified with the best jury model receiving a score between 0 (non-binding) and 1 (binding) regarding MHC-I H-2K^b^ binding potential.

The heptapeptide fragment-containing groups (xIINFEKL, SIINFEKx) comprising 20 peptides each exhibited the highest score distributions (*median values* = 0.99). In contrast, all trimer fragment-containing groups (xxxxxEKL, xxxxFEKx, xxxNFExx, xxINFxxx, xIINxxxx, SIIxxxxx) showed a median at the other end of the prediction range (0 to 0.03) with merely the xxINFxxx group exhibiting values distributed more dispersedly over the entire range [0,1[.

Tetrapeptide fragment-containing groups were categorized into low median groups xxxNFEKx, xxINFExx and SIINxxxx (0.01), and in opposition high median groups xIINFxxx (*median* = 0.75) and xxxxFEKL (*median* = 0.98).

Pentapeptide-fragment containing groups were divided into a high median category containing xxxNFEKL (0.98) as well as SIINFxxx (0.99), and the low median groups xIINFEx (0.16) and xxINFEKx (0.02), while the hexapeptide fragment-containing groups all exhibited high medians: xIINFEKx (0.95), SIINFExx (0.98), and xxINFEKL (0.99).

Examining groups with the same first non-arbitrary (non ‘*x*’) residue from the amino-terminal tail revealed a decrease of median predictions scores, when randomizing an increasing number of amino acids from the carboxy-terminus (*e.g.*, xxINFEKL, xxINFEKx, xxINFExx, xxINFxxx). However, upon randomizing glutamine at position 6 (E to x) this tendency was perturbed by a slight median increase. *Vice versa* held true for the amino-terminus with a similar yet weaker perturbation observed for randomization of the isoleucine at position 3 (*e.g.*, SIINFEKx, xIINFEKx, xxINFEKx, xxxNFEKx, xxxxFEKx). In general, randomizing amino acids at the main anchor positions 5 and 8, which in the case of SIINFEKL comply with the canonical sequence motif, always led to a decrease of the median score. The same held true for randomization of isoleucine at position 2, underlining a similar importance as the canonical anchors.

### Direct-binding experiments

**Figure 6:**
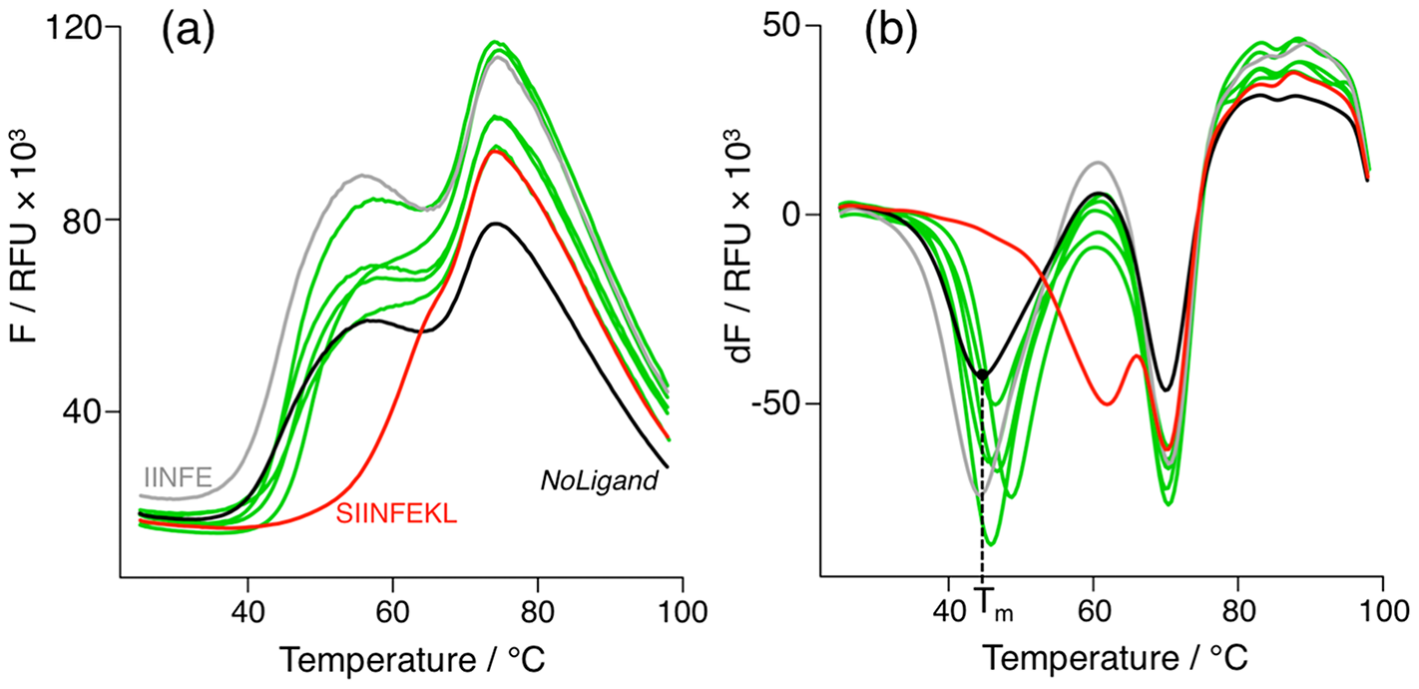
(**a**) Melting curves of peptide-MHC-I (H-2K^b^:IgG fusion protein) complexes depicting the normalized fluorescence *F* in relative fluorescence units (RFU) for SIINFEKL and *NoLigand* as positive (red line) and negative (black line) controls, an exemplary epitope fragment (INFE) showing no melting point shift (grey line) and all epitope fragments (IINFEKL, SIINF, INFEKL, SIINFEK, SIINFE) leading to a significant melting point shift (green lines). (**b**) Analogously the first derivative of *F* (d*F*) reveals the melting points as local minima, with *T*_m_ denoting the presumable MHC-I heavy chain melting point in the absence of peptide ligand *(NoLigand)*.

To test the predictions made by our machine-learning model, we synthesized all SIINFEKL fragments and measured MHC-I H-2K^b^ binding in a thermal denaturation assay (Fig. 6). The quadruplicate measurements exhibited high consistency between predicted and measured binding, indicated by low standard deviation for all peptide fragments with mean relative binding values greater than zero (Fig. 5b). Based on the unpaired Welch-test [28], the reference binder SIINFEKL showed a significant increase in binding (100%, *p*<9.9·10^−12^) in comparison to the unloaded MHC-I complex (denoted as *NoLigand* in Table 1). The amino-terminal serine-deprived heptapeptide IINFEKL showed the highest remainder of relative binding potential (22%, *p*<9.9·10^−8^). The majority of the remaining fragments showed no significant relative binding. Exceptions include the pentapeptide SIINF (*p*<0.002) and the hexapeptide INFEKL (*p*<0.003) exhibiting 8% respectively 7% relative binding on average, followed by the lowest yet still significant binding of SIINFEK (5%, *p*<0.001) and SIINFE (4%, *p*<0.002).

**Table 1:** Significance analysis examining quadruplicate binding measurements of SIINFEKL fragments.

Peptide	*p*-value <	Relative binding *mean* (σ)
EKL	0.9	-
FEKL	0.5	-
FEK	1	-
NFEKL	1	-
NFEK	0.9	-
NFE	0.9	-
INFEKL	0.001	7% (2%)
INFEK	0.4	-
INFE	1	-
INF	0.7	-
IINFEKL	0.001	22% (1%)
IINFEK	0.8	-
IINFE	0.9	-
IINF	0.9	-
IIN	0.5	-
SIINFEK	0.001	5% (1%)
SIINFE	0.001	4% (0%)
SIINF	0.001	8% (3%)
SIIN	0.9	-
SII	0.9	-
SIINFEKL	0.001	100% (1%)
*NoLigand*	0.5	-

The null hypothesis for the Welch-test [28] considers the distribution of respective peptide fragments measurements to not be greater than the distribution of *NoLigand* measurements.

It is noteworthy that the pentapeptide SIINF (8%) exhibited significantly higher (*p*<0.037 and *p*<0.014, respectively) binding compared to its elongated derivatives SIINFEK (5%) and SIINFE (4%). A potential explanation is provided by acknowledging glutamate as a suboptimal solution at position 6 (as reflected in the prediction score analysis), thereby neutralizing and even reverting the slightly positive effect on binding by solvent-interacting lysine in position 7. Though, it remains questionable how the pentapeptide SIINF lacking the C-terminal anchor backbone and side-chain interactions is able to induce the formation of the presumably highly flexible MHC binding cavity [1], [29], especially as the pentapeptide NFEKL containing both main anchors did not exhibit significant binding. Alternatively, analyzing a co-crystal structure of SIINFEKL in the MHC-I H-2K^b^ binding cleft (Fig. 1) reveals a compartmentation of the peptide-binding cavity into a small sub-pocket referred to as F-pocket [30], including the solvent accessible residues 6–7 as well as the C-terminus 8, and a large sub pocket from positions 1 to 5, which is presumably addressed by the SIINF fragment. It is conceivable that the SIINF fragment bound to the MHC protein solely stabilizes the larger sub-pocket by an induced flexible fit. Overall, only fragments exclusively retaining the C- or N-terminus of the fragment source showed significant albeit weak remaining binding potential with at best five-fold lower binding than the positive reference binder. The minimum required length to measure a significant binding potential was provided by the pentapeptide SIINF.

## Discussion

The results of this study demonstrate the potential of coupling cascaded machine-learning models for predicting MHC-I antigen presentation to a rapid thermal denaturation assay for validation of direct binding to MHC. Even more, implicating from prediction distributions of SIINFEKL-fragment containing peptides to direct-binding measurements of actual fragments appears to be feasible. The newly established model allows for a fine-grained grasp of sequence motifs, suggesting that sequence length, as defined in previous studies by an optimum of 8–10 amino acids for the H-2K^b^ allele, plays a crucial role in the binding mechanism [7], [31], [32]. Shorter fragment lengths or randomization of more positions led to lower prediction scores and decreased relative binding with a minimal but still significant effect exhibited by a pentapeptide. The results confirm the importance of serine respectively leucine as N- and C-termini for the example of ovalbumin-derived SIINFEKL [33], [34], as randomization of these positions concludes to substantially lower scores and lower relative binding measurements (Fig. 5). Comparison of relative binding decrease upon removal of C-terminal leucine reveals this peptide terminus as a dominant contributor even known to allow for longer peptides extending beyond the F-pocket [35]. The superior importance of the C-terminus is explainable by backbone and aliphatic side chain interactions as an anchor residue.

Concerning the classic canonical sequence motif (Fig. 1) [10], SIINFEKL main anchors (phenylalanine at position 5 and leucine at position 8) were validated by predictions as well as testing, though indication of an aliphatic secondary anchor at position 2 is suggested by observing a reduction of relative binding from IINFEKL (22%) to INFEKL (7%) and concurring predictions. The known secondary anchor at position 3, not fulfilled by the isoleucine of SIINFEKL, is confirmed by this residue's negative effect on binding. Furthermore a distinct negative effect of binding can be concluded for glutamate at position 6 (SIINFEK 5%, SIINFE 4%, SIINF, 8%), as albeit sequence length is decreased, relative binding increased after glutamate removal. Thus, the prediction model successfully captured both negative effects. In general, the model delivers a more fine-grained and differentiated perspective on the entire H-2K^b^ binding motif than the classic canonical motif, by not only focusing on anchor residues [36].

A further prospective analysis of the entire slice-and-diced mouse proteome revealed about 1.75% of octapeptides as confidently (*prediction score* ≥ 0.99) classified MHC-I H-2K^b^ binders. For successful rational vaccine designs, machine-learning models with sophisticated meta-learning schemes such as cascaded models should in future be trained for various alleles, rapidly scanning pathogen proteomes for MHC-I ligands. In fact, recent comparison of publicly available prediction tools and community benchmark studies revealed machine-learning models as state-of-the-art for MHC-I and MHC-II prediction, outperforming motif- and matrix-based models [37], [38]. We used a neural network as jury classifier, because ANN have proven to exhibit among the highest prediction accuracies (above 80% for a majority of alleles) [39], [40], having been successfully applied and experimentally validated for MHC-I [41] MHC-II [42] ligands. Several prominent MHC prediction tools utilize ANN algorithms, among them NETMHC [43] and MULTIPRED [44].

Ensemble models have shown their general usefulness in increasing predictive performance in comparison to their individual base classifiers [45], [46], with special emphasis on cascaded or stacked generalization architectures exhibiting significantly increased generalization potential [47]. As missing out epitopes as a consequence of false-negatives can be detrimental in perspective of reverse vaccinology, *i.e.* scanning and predicting entire pathogenic proteomes, and false-positives undoubtedly lead to increased experimental costs, optimizing the predictive performance with ensemble models can be the critical lever for successful epitope and vaccine design. To best of our knowledge, Hiss *et al.* proposed the only other cascaded model for MHC-I ligand prediction as a scoring function in the scope of agent-based exploration of sequence space [12]. This model incorporated only three base classifiers with three different descriptors and one learning scheme (ANN) propagating to a single jury neural network. For a recent review on computational resources for MHC ligand prediction, see Koch *et al.* [48].

It must be kept in mind though that for some pathogens, an adequate immune response may require activation of not only the cell-mediated MHC-I supported CD8^+^ T cell response but also the assistance of MHC-II facilitated CD4^+^ T cell responses or antibody-driven humoral responses [49]. Practical and theoretical difficulties when using synthetic linear peptides arise when considering this aspect [6]. In perspective of combatting pathogens or pathogenic components in extracellular spaces, activation of B cells for stimulation of antibody production requires antigen recognition by B cell receptors (BCR). BCRs exclusively specialize in the recognition of antigens located in specific conformations at pathogenic protein or toxin surfaces [50]. These antigens, also referred to as ‘neutralizing epitopes’, can rarely be mimicked by short linear synthetic peptide sequences as those investigated in this project [51]. Approaches to counteract this issue include usage of appreciably longer or cyclized peptides able to adopt a defined conformational ensemble [29] or structure-based approaches [52] trying to replace the peptide scaffold. We also wish to point out that the linear peptides focused on in this project can pose putative epitopes for priming of CD8^+^ cytotoxic T lymphocytes, while others, subject to MHC-II presentation, may prime CD4^+^ helper T cells (T_H_) [53]. Eventually the primed T cells will differentiate into armed effector and memory cells, providing not only acute but also long-term mediated recruiting of other immune agents by cytokine and chemokine secretion (T_H_) and defense against intracellular pathogen infection by induction of apoptosis [54].

## Materials and Methods

### Amino acid descriptors

**PEPCATS** is based on the CATS (*Chemically Advanced Template Search*) [55] topological pharmacophore representation of druglike molecules. Here we employed this concept to encode peptides in terms of their side-chain functionalities [56]. Instead of typing atoms, up to six different potential pharmacophore features are assigned to the amino acid residues (L: Lipophilic, R: Aromatic, A: Acceptor, D: Donor, P: Positive, N: Negative), which results in 21 different feature pairs (AA, AL, AR, *etc.*). Distances, in terms of sequence position, between two potential pharmacophore points of a respective residue pair are counted and binned for all pairs within a user-defined distance range. We used distances up to seven residue positions, yielding a 21×7 = 147-dimensional length-independent peptide descriptor. The resulting vector elements were scaled by dividing each value by the sum of all occurrences of the same feature pair type [57].

**PPCA** [58] represents peptides in terms of their physicochemical properties. Each amino acid in a peptide sequence is represented by 19 real-numbered values, which we obtained from a principal component analysis (PCA) performed on 143 acid property scales collected by Tomii and Kanehisa [59]. The first 19 principle components already account for about 100% of the variance, accordingly this results in a 20-residue×19-score matrix. Raw scores were scaled by unit variance, whereupon each row of the PCA score matrix corresponds to a 19-dimensional description of the respective amino acid.

**PPCALI** is a length invariant auto-correlated version of the PPCA descriptor. At first the PPCA vector is calculated and transformed into a matrix column-wise containing the 19 values for each amino acid of a dedicated peptide sequence. Hence this matrix is auto-correlated by summing up the products of matrix row elements with a defined correlation distance for every row, resulting in a total of 19 sums. This is performed for a user-defined correlation distance range (here: zero to seven residue positions). In this case the total dimension of the descriptor is given by concatenating 19 sums from correlation distance 0 to 7 equates to 152 dimensions.

**AAFREQ** is a 20-dimensional descriptor containing the amino acid frequencies as integers of a peptide sequence in a fixed order (A, R, N, D, C, Q, E, G, H, I, L, K, M, F, P, S, T, W, Y, V).

**BINAATYPE** represents each amino acid by six bits, whereas every bit encodes for the presence or absence of a certain pharmacophore feature (Lipophilic, Aromatic, Acceptor, Donor, Positive, Negative) [60]. BINAATYPE is based on the same pharmacophore types as PEPCATS.

**BINPEP** represents the identity of each amino acid by a string of five bits.

### Machine-learning

We used WEKA [61] for ANN and SVM classifier training, with the LIBSVM [62] C-support vector classification (C-SVC) wrapped by the respective Java classes for training the SVM classifiers.

### Thermal denaturation assay

Thermal denaturation studies were conducted using a StepOnePlus real-time PCR system (Applied Biosystems) with MicroAmp optical 96-well plates (Applied Biosystems cat. no. N8010560). Wells were loaded with 10 µl of H-2K^b^:IgG fusion protein solution (protein conc. = 0.5 mg×ml^−1^; BD Biosciences cat. no. 550750) – respectively 10 µl of PBS buffer (pH 7.36) for ligand-only controls – 2 µl of peptide or peptide fragments, 2 µl of 10× SYPRO Orange (SigmaAldrich cat. no. S5692) and 6 µl (8 µl for negative controls) to yield a total volume of 20 µl per well. Final concentrations calculated to 1 µM for H-2K^b^:IgG fusion protein, 100 µM for the peptides/peptide fragments and 1× SYPRO Orange. Fluorescence intensity was measured using the Applied Biosystems ROX preset with respective excitation/emission maxima at 587/607 nm, while heating the wells continuously from 25°C to 99°C with a ramp rate of 1% (temperature increase of 1.5°C per minute). Results were recorded by StepOne 2.2.2 software and analyzed by identifying the local minimum of the derivative of the melting curve for the segment relevant for denaturation of the peptide-binding superdomain (α_1,2_) of the MHC-I heavy chain.

### Peptide synthesis and analytics

DMF (dimethylformamide), DCM (dichlormethane), diisopropylether, piperidine and TIPS (triisopropylsilane) were purchased from Sigma-Aldrich. NMM (4-methylmorpholine) and TFA (2,2,2-trifluoroacetic acid) were acquired from Fisher Scientific; Fmoc-protected Wang-resins, Fmoc-protected amino acids, and HCTU (*O*-(6-chloro-1-hydrocibenzotriazol-1-yl)-1,1,3,3-tetra -methyluronium hexafluorophosphate) were obtained from AAPPTEC. An Overture robotic solid phase peptide synthesizer (Protein Technologies, Tucson, USA) was used to synthesize peptides on a 20 µmol scale utilizing 10-fold excess of Fmoc-protected amino acids (200 mM) relative to the Fmoc-Wang-resin. Deprotection by 20% piperidine in DMF for 2×5 min. Double coupling was performed in a conservative manner of 2×15 min rotations with a ratio of 1∶1∶2 (200 mM amino acid, 200 mM HCTU, 400 mM NMM) in DMF. DMF washing was applied for 5×30 sec after deprotection and double coupling. Automated cleavage was applied for 2 h with 95% vol.+2.5% vol.+2.5% vol. (TFA, H_2_O, TIPS), after multiple washing steps with DCM (3×30 sec). Using ice-cold diisopropylether, the peptides were precipitated from the final TFA-peptide solution and rewashed. All peptide products were analyzed on an LC-20A HPLC instrument (Shimadzu) using an rpC18, 110 Å, 5 µm, 150×3 mm column (Macherey-Nagel), with a linear gradient of 5–70% ACN/H_2_O (0.1% TFA) over 25 min with a flow rate of 0.5 ml×min^−1^; UV-VIS detection at 210 nm. Masses were detected between 300–1500 Da with a Shimadzu LCMS-2020 single-quad mass spectrometer (ESI+). UV^210^ purity of all products: >98%. Peptide sequences are denoted with the calculated molecular weight (*mw*, unit: Da), observed retention times (*Rt*, unit: minutes) and masses (*m*+); some peptides exhibited water clusters (*): SII (*mw* = 331.40, *Rt* = 9.87, *m*+ = 332.15, 333.15, 663.4, 664.4), IIN (*mw* = 358.43, *Rt* = 6.86, *m*+ = 359.15, 360.15, 719.40, 717.45, 718.45), INF (*mw* = 392.45, *Rt* = 10.15, *m*+ = 393.15, 394.10, 786.30, 785.4, 1175.75), NFE (*mw* = 408.41, *Rt* = 7.66, *m*+ = 408.10, 409.95, 817.35), FEK (*mw* = 422.48, *Rt* = 6.45, *m*+ = 432.20, 424.20), EKL (*mw* = 388.46, *Rt* = 7.59, *m*+ = 389.15, 390.15, 777.45), SIIN (*mw* = 445.51, *Rt* = 8.86, *m*+ = 446.25, 447.25, 891.55, 892.50), IINF (*mw* = 505.61, *Rt* = 10.36, *m*+ = 527.35*, 528.25*, 529.30*, 957.30*, 958.40*), INFE (*mw* = 521.57, *Rt* = 9.77, *m*+ = 522.25, 523.20, 1043.55, 1044.55, 1045.50), NFEK (*mw* = 536.59, *Rt* = 6.93, m+ = 537.25, 538.25, 539.25), FEKL (*mw* = 534.63, *Rt* = 9.39, *m*+ = 536.35, 537.25, 538.20, 1071.65, 1072.60), SIINF (*mw* = 592.68, *Rt* = 11.99, *m*+ = 593.30, 594.35, 596.25, 1184.85, 1185.75, 1186.70, 1187.70), IINFE (*mw* = 634.72, *Rt* = 10.76, *m*+ = 635.35, 636.30, 637.30, 1269.70, 1270.80), INFEK (*mw* = 649.74, *Rt* = 8.84, *m*+ = 325.75, 650.35, 651.35, 652.35), NFEKL (*mw* = 649.74, *Rt* = 9.59, *m*+ = 650.35, 651.35, 652.30, 1300.85), SIINFE (*mw* = 721.79, *Rt* = 11.5, *m*+ = 722.40, 723.40, 724.30, 1443.9, 1444.90, 1445.90), IINFEK (*mw* = 762.89, *Rt* = 9.77, m+ = 382.35, 763.40, 764.40, 765.40), INFEKL (*mw* = 762.89, *Rt* = 10.84, *m*+ = 382.30, 763.45, 764.4, 957.45, 965.45, 965.45), SIINFEK (*mw* = 849.97, *Rt* = 10.51, *m*+ = 425.80, 850.50, 851.45, 852.45), IINFEKL (*mw* = 876.05, *Rt* = 11.51, *m*+ = 438.95, 876.55, 877.50, 878.50).
